# Mechanistic insights into the efficacy of cell penetrating peptide-based cancer vaccines

**DOI:** 10.1007/s00018-018-2785-0

**Published:** 2018-03-05

**Authors:** Morgan Grau, Paul R. Walker, Madiha Derouazi

**Affiliations:** 1 Amal Therapeutics, Geneva, Switzerland; 2 Center for Translational Research in Onco-Hematology, Division of Oncology, Geneva University Hospitals and University of Geneva, Geneva, Switzerland

**Keywords:** Cell penetrating peptides, Cancer vaccines, Immunotherapy, Tumor antigens, Antigen processing

## Abstract

Immunotherapies are increasingly used to treat cancer, with some outstanding results. Immunotherapy modalities include therapeutic vaccination to eliminate cancer cells through the activation of patient’s immune system against tumor-derived antigens. Nevertheless, the full potential of therapeutic vaccination has yet to be demonstrated clinically because many early generation vaccines elicited low-level immune responses targeting only few tumor antigens. Cell penetrating peptides (CPPs) are highly promising tools to advance the field towards clinical success. CPPs efficiently penetrate cell membranes, even when linked to antigenic cargos, which can induce both CD8 and CD4 T-cell responses. Pre-clinical studies demonstrated that targeting multiple tumor antigens, even those considered to be poorly immunogenic, led to tumor regression. Therefore, CPP-based cancer vaccines represent a flexible and powerful means to extend therapeutic vaccination to many cancer indications. Here, we review recent findings in CPP development and discuss their use in next generation immunotherapies.

## Introduction

Cell penetrating peptides (CPPs) are peptides of 8–40 residues that have the ability to cross the cell membrane and enter into most cell types. Alternatively, they are also called protein transduction domain (PTDs) reflecting their origin as natural proteins. It is now more since 20 years since Frankel and Pabo as well as Green and Lowenstein described the ability of the trans-activating transcriptional activator from the human immunodeficiency virus 1 (HIV-TAT) to penetrate into cells [1]. In 1991, transduction into neural cells of the Antennapedia homeodomain (DNA-binding domain) from *Drosophila melanogaster* was described [2]. In 1994, the first 16-mer CPP called Penetratin (RQIKIYFQNRRMKWKK) was characterized from the third helix of the homeodomain of Antennapedia [3], followed in 1998 by the identification of the minimal domain of TAT (YGRKKRRQRRR) required for protein transduction [4]. More recently, other peptides of different origin were described; these include viral proteins (e.g., VP22 [5] and ZEBRA [6]), or those present in venoms (e.g., melittin [7], mastoporan [8], maurocalcin [9], crotamine [10] or buforin [11]). Synthetic CPPs were also designed including the poly-arginine (R8, R9, R10 and R12) [12] or transportan [13]. All of these CPPs can be classified into three different categories: (1) cationic CPPs such as TAT, penetratin, and poly-arginine, in which the positive charge relies principally on arginine and lysine residues; (2) amphipathic CPPs such as Transportan and Pep-1, where the cationic residues are separated by hydrophobic residues and the positive charge relies principally on lysine residues; (3) hydrophobic CPPs [14]. The different classes of CPPs have been extensively reviewed elsewhere [14].

Over the past 20 years, multiple applications and uses of CPPs have been described. These very versatile and promising vectors were used for intracellular delivery of a wide range of cargos, such as small molecules, nucleic acids, peptides and proteins. In many cases this is in the context of drug delivery in the field of oncology. In this review, we will describe applications of CPPs to cancer immunotherapy, with particular emphasis on cancer vaccines. To understand the rationale and the critical factors for efficacious use, we will discuss several important notions, such as the internalization route followed by the CPP-cargo, the nature of antigenic cargos, and the choice of adjuvants.

## CPPs coupled to antigenic cargos and their application to cancer vaccines

Cell penetrating peptides are well-suited to deliver antigenic peptides or proteins to induce adaptive immune responses [15]. Although most vaccines for infectious diseases also achieve this, the response of protein or inactivated viral vaccines is biased towards CD4 T cells and neutralizing antibodies. For cancer vaccines, induction of an integrated immune response including a CD8 T-cell component is considered essential. Dendritic cells (DCs) are professional antigen presenting cells (APCs) able to activate both CD8 and CD4 T cells by presenting captured-Ag in association with MHC-I or MHC-II molecules, respectively; CPPs can facilitate this key immunological mechanism [15]. We demonstrated the feasibility of this approach using a CPP (Z12) derived from EBV ZEBRA protein linked to a long OVA derived peptide containing the CD8-specific OVA_257–264_ epitope [16]. Vaccination of mice with a very low dose (10 nM) of this construction and (adjuvanted with anti-CD40 antibody and Hiltonol) led to the generation of a strong OVA-specific CD8 T-cell response, as revealed by OVA_257–264_ dextramer staining. In contrast, when vaccination was undertaken with the same dose of OVA long peptide antigen without CPP, immune response induction was negligible. OVA-specific CD8 T-cell immune responses have also been observed by linking the model ovalbumin to other CPPs such as TAT [17, 18], Penetratin [19, 20] and the translocation motif of HBV [21]. Moreover, immunization with TAT fused to OVA or HPV-E7 resulted in long-term protection in tumor re-challenge experiments, indicating memory response induction [22, 23].

Eliciting immune responses to tumor-associated Ag (TAAs) that are also self Ag is particularly challenging, since such Ag are generally poorly immunogenic due to central tolerance. We showed that vaccination of mice with Z12 or other ZEBRA-derived CPPs (Z13 and Z14) linked to the gp100 TAA/self Ag led to the generation of a gp100-specific CD8 T-cell response [16, 24]. Accordingly, we observed that Z14-gp100 therapeutic vaccination of mice injected iv with B16 cancer cells expressing gp100 leads to a near twofold reduction in the number of detectable lung metastases. In the same experimental system, we measured more than threefold reduction of lung metastasis in mice vaccinated with Z13 linked to another TAA/self Ag, TRP2 [24]. These series of observations demonstrate that the ZEBRA-derived CPP-linked to antigenic cargo is a very powerful system able to break self-tolerance and to induce therapeutic anti-tumor immune responses in vivo. Increased Ag-specific immune responses have also been reported by linking other tumor Ag to a CPP, such as TRP2 [25–27], carcinoembryonic antigen (CEA) [28], p53 [29], survivin [30], MUC-1 [31], HPV16 E7 [23], or HER2/neu [32]. Moreover, vaccination with these constructs induced either prophylactic or therapeutic anti-tumor effects in vivo. Considering that these Ag are often less immunogenic than model antigens such as OVA, all these observations support the use of CPPs as cancer vaccine vectors. One potential mechanism that can explain the efficacy of CPP-based cancer vaccines might be increased presentation of antigenic peptides on MHC molecules on the surface of APCs. In this regard, Wang et al. demonstrated that linking TRP2 Ag to CPP1 prolongs Ag presentation by DC [33]. Indeed, DC transduced with CPP-TRP2 stimulated TRP2-specific T cells in vitro for much longer periods than TRP2-pulsed DC. Another potential explanation would be that unlike protein-based vaccines, CPP-based vaccine might be more efficient at targeting of Ag processing and presentation machinery.

## Antigen-presentation pathways followed by CPP-Ag cargos

**Fig. 1:**
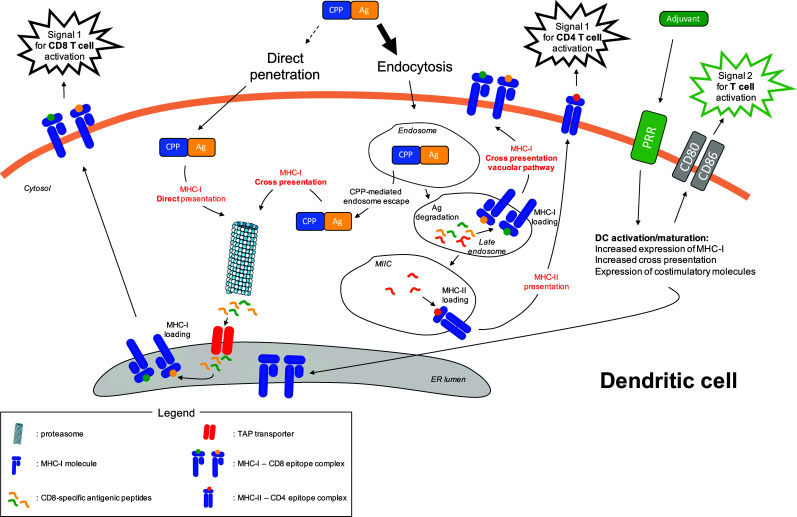
Ag processing and presentation pathways followed by CPP-Ag in dendritic cells. CPP-Ag mainly enters into the cells via endocytosis, although minor direct penetration across the plasma membrane cannot be ruled out. In the latter situation, antigenic cargo follows the MHC-I direct presentation pathway. Cytosolic antigenic cargo is processed by the proteasome into small antigenic peptides. The transporter associated with antigen processing (TAP) transfers these peptides into the endoplasmic reticulum (ER) where they are loaded onto MHC-I molecules. These complexes are transported to the plasma membrane and deliver the first signal for CD8 T-cell activation through their highly specific interaction with the T-cell receptor (TCR) of cognate CD8 T cells. Endocytosed CPP-Ag can be directed to different pathways. The MHC-I cross presentation pathway is reached by the antigenic cargo after its escape from the endosome due to the intrinsic properties of CPPs. Alternatively, the antigenic cargo can be degraded into small antigenic peptides in the endosome. As MHC-I molecules can be expressed in late endosomes (see Ref. [36]), some of these antigenic peptides can be loaded on these molecules, through the MHC-I cross presentation vacuolar pathway. Antigenic peptides also reach the MIIC compartment where they are loaded on MHC-II molecules that are then transported to the plasma membrane. These complexes deliver the first signal for CD4 T-cell activation through their highly specific interaction with the TCR of cognate CD4 T cells. The role of adjuvant is also depicted. It triggers pattern recognition receptors (PRR), leading to the activation and maturation of dendritic cells. In particular, adjuvant increases the MHC-I cross presentation activity of dendritic cells and also induce the expression of costimulatory molecules that are essential for T-cell activation through the delivery of costimulatory signals. Templates from Servier Medical Art image bank were used to draw this figure

Although some direct plasma membrane penetration can occur, especially at the high CPP concentrations reached in some experimental systems [34], it has been demonstrated that the main translocation mechanism used by CPP-cargo constructions is the endocytic pathway (for review, see Ref. [35]) (Fig. 1). In the context of therapeutic cancer vaccines, the mechanism of CPP-cargo entry plays a central role for antigen delivery and subsequent presentation. Cytosolic proteins will be processed and presented on MHC class I molecules, whereas exogenous proteins taken up by endocytosis will be processed and presented by MHC class II molecules (Fig. 1). However, certain DC subsets can also cross present exogenous Ag on MHC-I molecules [36]. To ensure efficient MHC-I presentation of CD8-specific TAAs, antigenic cargos linked to CPP and trapped in endosomes have to reach the cross presentation pathway.

Classical cross presentation involves a proteasome-dependent Ag degradation step that occurs in the cytoplasm. Thus, if Ag enter the endosomes, they must subsequently escape to enter this cross presentation pathway. Indeed, DC subsets can release endosome-trapped Ag into the cytosol, a process thought to involve the recruitment of endoplasmic reticulum (ER)-molecules, such as SEC61 and p97 ERAD proteins, to the endosome compartment [36]. However, it was demonstrated that certain CPPs can promote Ag endosome escape (Fig. 1). By comparing three CPPs linked to OVA, Mitsui et al. showed that the R9-OVA cancer vaccine is efficient at inducing OVA-specific immune responses in vitro and in vivo, leading to a strong anti-tumor effect [37]. When coupled to OVA, the CPP LAH4 induces tenfold stronger OVA-specific OTI T-cell responses in vitro compared to other well-known CPPs including TAT [27]. This increased Ag presentation could be linked to the fact that LAH4 CPP changes its conformation upon endosome acidification, leading to a facilitated release of antigenic cargo in the cytosol [38]. The CPP KALA adopts an alpha helical conformation at physiological pH (7.4), enabling it to perform membrane destabilization [39]. Taking advantage of this property, Miura et al. showed that OVA-liposomes fused to KALA exhibited increased MHC-I restricted Ag presentation compared with OVA-liposomes fused to the highly efficient transducer R8 CPP [40]. As a result, OVA-liposome-KALA vaccination induced higher in vivo CD8 T-cell responses, as revealed by an in vivo cytotoxic assay, and stronger anti-tumor effect in a prophylactic vaccination protocol using EG7 tumor challenge. Surprisingly, an acidic pH from 6.5 to 5.5 strongly increased the OVA-liposome-KALA fusogenic activity, suggesting that this particular construct might destabilize endosome membranes. GV1001 is a telomerase-derived peptide initially used as an MHC class II-restricted epitope in cancer vaccines. Surprisingly, this peptide induced strong in vivo CD4 and CD8 T-cell responses. This unexpected feature relies on the particular cell penetrating property of GV001 enabling it to reach the cross presentation machinery. Unlike TAT or other CPPs, the GV1001 cell penetration mechanism involves interaction with extracellular heat shock proteins 70 and 90, leading to accumulation of this CPP and its cargo in the cytoplasmic compartment [41].

Several studies showed that CPP could be tailored to favor cargo endosome escape. The HA2 domain of influenza haemaglutinin is a pH sensitive membrane disruptive peptide. Using a TAT-Cre GFP-flox reporter assay, Wadia et al. showed that linking HA2 to the TAT CPP (TAT-HA2) increased the GFP signal observed in transduced cells compared to TAT, due to increased endosomal escape of the CPP-cargo [42]. Similarly, including 10 Histidine moieties to the TAT CPP led to increased endosome escape of the cargo through increased endosome membrane disruption [43]. Finally, Mae et al. showed that N-terminal stearylation of the transportan 10 CPP increased its endosome escape, while it had no effect on penetratin endosomal escape [44]. Regarding ZEBRA-derived CPPs, we used a live cell β-lactamase reporter assay to monitor cytosolic free protein; β-lactamase activity with Z12-36KDa was detected with cargo concentrations as low as 100 nM, indicating that Z12 efficiently entered the cytosol, although the mechanism was not defined [16].

An alternative vacuolar cross presentation pathway has been described in DCs, which does not require Ag escape from endosomes [36]. In this vacuolar cross presentation pathway, both Ag degradation and epitope loading on MHC-I occur within the endosome and are independent of the proteasome and TAP transporter, two key players in conventional cytosolic cross presentation pathway (Fig. 1). Moreover, it has been demonstrated that protein degradation in endosomes is less efficient in DC compared to other phagocytic cells [45]. Accordingly, several studies reported that CPP-linked Ag processing is TAP- and proteasome-independent, suggesting that the vacuolar cross presentation pathway might also be used [46, 47].

While the complete mechanism of CPP-linked Ag processing and presentation is not fully understood, many of the proof of concept in vivo studies mentioned above showed that the CPP antigenic cargo is efficiently targeted to the MHC-I cross presentation pathway. Indeed, the best readout for efficient targeting of MHC-I cross presentation is the induction of strong Ag-specific CD8 T-cell responses in vivo. Nevertheless, in the context of CPP-based cancer vaccine development, this targeting can and must be optimized to ensure maximal Ag presentation by DCs to T cells.

## Multi-epitopic antigenic cargos

**Fig. 2:**
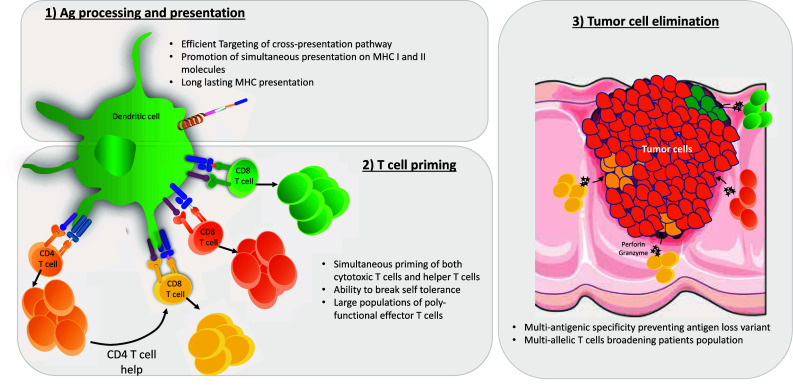
CPP-based cancer vaccines elicit powerful anti-tumor T-cell responses. CPP vectors allow targeting of multi-epitopic antigenic cargos to processing and presentation machinery for both MHC-I and MHC-II. In the context of appropriate adjuvant, transduced dendritic cells are then able to strongly activate both CD8 and CD4 T cells specific for multiple tumor-derived antigens. The resulting poly-functional effector T-cells migrate to the tumor site, where they eliminate heterogenous tumor cells via cytotoxicity. For each step, the main advantages of CPP-based vaccines are indicated. Templates from Servier Medical Art image bank were used to draw this figure

An important characteristic of a CPP-based cancer vaccine is the diversity of its antigenic cargo. Indeed, targeting multiple Ag has several benefits (Fig. 2). First, it might limit tumor escape from immune system pressure through the phenomenon of antigen loss variants [48]. Second, it might enlarge the spectra of cancer cells targeted within a single tumor. Indeed, most tumors exhibit heterogeneous Ag expression [49]. Finally, it might enable the same vaccine to be used for wider patient groups by providing epitopes restricted by multiple HLA alleles. We constructed such a multi-epitopic antigenic cargo, including 3 CD8 and 2 CD4-specific epitopes, and linked it to the Z12 CPP. In vitro, bone marrow-derived DCs transduced with low amounts (0.3 µM) of this construction efficiently stimulated monoclonal CD8 and CD4 T cells of different Ag-specificities; this indicated that each epitope of the cargo was efficiently processed and presented by DCs. In a more physiological polyclonal setting, we demonstrated that vaccination of mice with low amounts of this construct (10 nmol) together with adjuvants induced T-cell immune responses for each of five epitopes, as revealed by MHC-dextramer staining or intracellular cytokine staining [16]. In other studies, TAT was also used as vector for a multi-epitopic cancer vaccine [50, 51]. Multi-epitopic presentation was detected in vitro with 3 µM peptide and in vivo with 100 µmol of peptide when furin-sensitive spacers were added between the epitopes [50, 51]. HSPs derived from cancer cells can form complexes with multiple tumor-derived Ag that can be up taken by DCs to elicit tumor-specific T-cell immune responses. Nishikawa et al. demonstrated that linking HSP70 to the VP22 CPP dramatically enhances HSP uptake by DCs. Accordingly, in vivo intratumoral electroporation of plasmid encoding VP22-HSP70 in EG7 tumor bearing mice led to strong inhibition of tumor growth. Interestingly, CD8 T cells from vaccinated mice not only lysed OVA-expressing EG7 tumor cells, but also parental EL4 tumor cells that do not express OVA Ag. Thus, combining VP22 to HSP70 generated multi-epitopic CD8 T-cell immune responses in vivo [52].

Although CD8 T cells are important for the elimination of cancer cells by cytotoxicity, CD4 T cells are also required for efficacious anti-tumor immunity, through direct effector function, helper activity for CD8 T cells, and enhancement of effector cell infiltration at the tumor site [53]. Therefore, multi-epitopic antigenic cargos linked to CPP stimulating both CD4 and CD8 T cells might also be important to generate a fully functional anti-tumor immune response (Fig. 2). This was directly demonstrated using Zebra-derived CPPs, TAT, and penetratin [16, 22, 24, 46, 54, 55].

## Importance of adjuvants in CPP-based cancer vaccines

To be fully activated, T cells have to receive two main signals that are the antigenic signal (signal 1) and the costimulatory signal (signal 2). Moreover, the T-cell response is also shaped by a third signal mediated by cytokines. Signal 1 and signal 2 are delivered by activated mature DCs, as are many cytokines. Triggering of pattern recognition receptors (PRR) on DCs by DAMPs (danger associated molecular pattern) induces increased expression of MHC molecules, costimulatory molecules and cytokines [56] (Fig. 1). Although CPPs are sometimes considered as adjuvants for peptide-based cancer vaccines due to increased intracellular Ag delivery, transduction of immature DC with a CPP-Ag cargo did not induce DC activation and maturation [57–59]. Therefore, CPP-based cancer vaccines must be used in combination with adjuvants to elicit fully functional anti-tumor immune responses.

Several factors, such as limited depot effect and optimal immunopotentiator effect, have to be made regarding the choice of adjuvants used in combination with cancer vaccines [60]. Accordingly, we demonstrated that the choice of adjuvant directly impacts on the immune responses and subsequent anti-tumor effects elicited by CPP-based cancer vaccines. We vaccinated mice with ZEBRA-derived CPPs linked to OVA CD4 and CD8 specific Ag, in combination with Hiltonol (a TLR3 ligand), Pam3CSK4 (a TLR2 ligand) or MPLA (a TLR4 ligand). Efficacy of CPP-adjuvant combinations varied considerably when vaccine-induced CD4 and CD8 T-cell frequencies were measured, which translated into potent therapeutic effect for certain vaccines. Indeed, while all of the adjuvanted vaccines enhanced survival, that induced by Hiltonol or MPLA was particularly striking and more pronounced than that achieved with Pam3CSK4 adjuvant [24]. This study highlights that the choice of adjuvant is a crucial step in the development of a CPP-based cancer vaccine.

The negatively charged double stranded RNA Poly I/C (a less stable equivalent of Hiltonol) is easy to link to cationic CPPs through electrostatic interactions. Transduction of immature DCs with TAT linked to MelanA Ag and Poly I/C induced strong DC activation and maturation, as shown by up-regulation of CD83, CD86 and high production of IL-12 [58]. Vaccination of MC38-CEA tumor-bearing mice with TAT-CEA-poly-IC induced a strong decrease of tumor growth compared to TAT-CEA injected mice [57]. In a DC vaccination protocol, Mitsui et al. vaccinated EG7 tumor bearing mice with R9-Ova transduced DCs coupled with three injections of OK432 or LPS (two TLR4 ligands) as adjuvants. They observed that all mice vaccinated with CPP-transduced DCs and adjuvants completely rejected EG7 tumor compared to mice vaccinated with CPP-transduced DCs alone, demonstrating the strong additive effect of adjuvant when used with a CPP-based cancer vaccine [37]. In vivo injection of LAH4-protein with CpG (a TLR9 ligand) increased retention of cargo protein at the site of injection, protein transport to the draining lymph node, and T-cell activation. In a therapeutic vaccination protocol, LAH4-OVA-CpG induced a strong anti-tumor effect in B16-OVA tumor bearing mice compared to LAH4-OVA vaccinated mice, as shown by the very strong delay of tumor growth observed [27]. Similar results were observed in a prophylactic vaccination protocol using penetratin-OVA-CpG in a B16 tumor challenge [47].

Cytokines can also be used at adjuvants. In an in vivo vaccination protocol, an IL-15 encoding plasmid linked to TAT CPP and survivin epitope increased the percentage of survivin-specific CD8 T cells with lytic function towards CT26 tumor cells. Moreover, IL-15 adjuvant increased the generation of memory T cells. In CT26 tumor bearing mice, TAT-survivin-IL-15 vaccination was more efficacious than TAT-survivin vaccination [61]. Vaccination of mice with TAT-HPV E7 Ag coupled to a plasmid encoding GM-CSF induced stronger Ag-specific CD8 T-cell responses compared to TAT-E7. This prophylactic vaccination protocol provided a high level of protection, in which 90% of mice challenged with TC1 tumor cells remained tumor free. In a therapeutic vaccination setting, TAT-E7-GM-CSF provided a strong anti-tumor effect dependent on CD8 T cells and to a lesser extent on CD4 T cells. Moreover, vaccination with TAT-E7-GM-CSF induced more memory CD8 T cells than TAT-E7, conferring long lasting anti-tumor protection [23]. Interestingly, GM-CSF was shown to strongly promote the cross presentation of Ag by DCs [62–64]. Nevertheless, vaccination protocols need to be carefully optimized for this cytokine adjuvant, since either positive or negative effects were reported [65].

In addition to enhancing T-cell responses, adding adjuvants to CPP-based cancer vaccines might also be important to modify the immunosuppressive tumor microenvironment. Indeed, Spinetti et al. recently showed that injection of the TLR7 agonist R848 induced a strong decrease of intratumoral MDSC [66]. The monocytic MDSC subset was the most affected by this treatment. In addition, R848 blocked the immunosuppressive function of this cell population, reducing tumor progression. In a similar manner, injection of an engineered *Salmonella typhimurium* strain secreting *Vibrio vulnificus* flagellin B in MC38 tumor bearing mice induced a strong anti-tumor effect, in part through the reprogramming of tumor-associated macrophages to an M1-anti-tumoral phenotype [67].

## Route of administration of CPP-based cancer vaccines

The main drawback of CPP-based technology is its lack of cell or tissue specificity. As a consequence, systemic injection of CPP leads to integration in multiple cell types (epithelial cells, fibroblasts, leukocytes) within multiple organs [68, 69]. Strategies have been developed to confer some cell specificity to CPP-based treatments, including coupling to homing peptides or use of activatable CPPs (reviewed in [70]). Although efficacy has been demonstrated, these constructs are mainly used for direct targeting of cancer cells. To the best of our knowledge, no CPP targeting specific immune cell types such as DCs has been developed so far.

Given the crucial role played by DCs in the initiation of T-cell responses, it is of major interest to specifically target vaccine-associated antigens to this cell type. Some of these sentinel cells are positioned in various peripheral sites of the organism, such as skin and mucosal surfaces, where the probability to encounter pathogen is the highest. After Ag capture by these DCs, they start to mature while migrating to draining secondary lymphoid organs to present MHC–Ag complexes to naïve T cells, leading to their activation [71]. Thus, as for many conventional vaccines, CPP-based vaccines are administered through parenteral injection (either i.d. or s.c.) to maximize the probability of targeting DCs. This route of administration of CPP-based cancer vaccines has been proven to be efficacious, as revealed by strong Ag-specific T-cell responses elicited against various Ag [21, 24, 47, 72]. An elegant study of Zhang et al. clearly showed that s.c. administration of a CPP-based vaccine efficiently triggers peripheral DCs [27]. Subcutaneous injection of a fluorescent protein coupled to the LAH4 CPP led to increased retention of this protein at the injection site. Strong fluorescence was also detected in inguinal draining lymph nodes. Addition of CpG adjuvant to this CPP-based vaccine dramatically increased the fluorescence level observed in these lymph nodes. These observations suggest that s.c. injection of CPP-based vaccines efficiently targets skin-resident DCs. Addition of an adjuvant provides the danger signal essential for these targeted DCs to mature and migrate to draining secondary lymphoid organs where they can present captured-Ag to naïve T cells and thus initiate an Ag-specific T-cell response. Accordingly, stimulation of OTI T cells with inguinal lymph node cells from mice vaccinated s.c. with LAH4-OVA-CpG resulted in an almost twofold increase of INFγ producing OTI T cells compared to stimulation using lymph node cells from OVA-CpG vaccinated mice.

Using the ANTP CPP coupled to the CD8 epitope SIINFEKL, Schutze-Redelmeier et al. show that the epicutaneous route of vaccine administration induced the strongest SIINFEKL-specific T-cell response, compared to s.c. and i.p. administrations that did not generate detectable responses [73]. Nevertheless, this observation might be due to the fact that this vaccine, compared to the one used by Zhang et al., was administered without adjuvant. In the epicutaneous route, the use of multiple tape-stripping of the skin and acetone treatment might create a local inflammation compensating for this absence of adjuvant.

Other challenging administration routes have been explored to inject cancer vaccines. Intranodal injections of RNA-based cancer vaccines showed induction of very strong Ag-specific T-cell responses in mice [74]. However, we found that using this route of administration for a CPP-based therapeutic cancer vaccine generated an Ag-specific immune response of lower magnitude compared to the s.c. route (our unpublished observations).

Some studies suggest that certain immune cells are more prone to be targeted than others by CPP-based vaccines. Schwarze et al. show that in vivo injected TAT-β galactosidase construct preferentially accumulated in the red pulp of the spleen, while the white pulp remained largely β galactosidase negative [69]. An interesting recent study of Lim et al. shed light on a preferential uptake of CPP-based vaccines by phagocytic cells (DCs and macrophages) compared to other immune cells [75]. In vitro incubation of mouse splenocytes with EGFP coupled to TAT or dNP2 CPPs led to increased EGFP fluorescence in DCs and macrophages compared to lymphocytes. A similar observation was made on splenocytes from mice injected i.v. with these constructs. This preferential targeting of DCs and macrophages by CPP could rely either on their intrinsic phagocytic activity and/or on a different plasma membrane composition of these cells compared to other immune cells, such as heparan sulfate proteoglycans. These molecules are particularly important for the binding and internalization of at least some CPPs [76]. Thus, based on these observations and on the fact that skin is a DC-rich anatomic site, it appears that i.d./s.c. injection might be the most efficacious administration route for CPP-based cancer vaccines.

## CPP-based cancer vaccines in clinical trial

Many cancer vaccines are currently being tested in clinical trials. As DCs are key players in the induction of adaptive immune responses, autologous or HLA-matched Ag-pulsed DCs have been widely used to treat various types of cancers [77]. However, this process is expensive, and to date has offered only modest clinical benefit. Several factors, such as poor MHC class I targeting, short-lived Ag presentation (MHC turnover), or low density of TAAs can be attributed to explain this poor efficacy [78]. In contrast, as discussed above, CPPs represent an outstanding alternative to DCs as a cancer vaccine vector, by increasing Ag uptake, targeting both MHCI and II Ag presentation pathways, by increasing the duration of Ag presentation, and by increasing the magnitude of Ag-specific immune responses [33, 78]. Nevertheless, a prerequisite is the identification of antigens (TSA or TAA) that can function as tumor-rejection antigens. To date, only a few studies, to our knowledge, have used CPP as a vector for cancer vaccine delivery in a clinical setting. Gliknik Inc. developed a Trojan cancer vaccine, consisting of chemically synthetized long peptides, containing 2 or 3 CD4 and CD8 specific epitopes from either MAGE or HPV Ag, fused to the TAT CPP. A furin-sensitive spacer was added between the epitopes [79]. This approach is based on results showing induction of antigen-specific cytotoxic T lymphocytes after immunization with synthetic long peptides fused to the TAT. In a pilot study, vaccination of five patients suffering from head and neck carcinoma with Trojan vaccine coupled with Montanide and GM-CSF as adjuvants induced antigen-specific CD4 T cells and IgG antibodies. Interestingly, Ag-specific CD8 T-cell responses were poorly elicited, perhaps suggesting that antigenic cargo failed to reach the MHC-I cross presentation pathway with this vaccine. None of the patients developed an objective clinical response. Nevertheless, a phase I dose escalation trial was undertaken in a larger cohort of recurrent/metastatic head and neck squamous cell carcinoma patients using the same vaccines [80]. Consistent with their pilot study, induction of Ag-specific CD4 T cells and antibodies was observed in the majority of patients that were vaccinated 4 times. Again, Ag-specific CD8 T-cell responses were not detected. Based on RECIST criteria, no clinical benefit was observed, except for one patient among seven in the MAGE-A3 cohort who experienced stable disease for 10.5 months.

## Concluding remarks

Cell penetrating peptides are very promising tools for the development of new cancer therapies. Conjugating various toxic/antigenic cargos to these short peptides has proven efficacious in specifically targeting and eliminating cancer cells. In the field of cancer vaccines, pre-clinical as well as clinical studies have demonstrated that the use of CPPs as vaccine vectors represents an efficacious strategy to generate strong cancer-specific immune responses. Such an approach is particularly relevant in the case of so-called “cold tumors” that are known to be poorly immunogenic. However, it is challenging to induce an immune response against weakly immunogenic tumors, particularly if the target antigens are TAA to which there is partial immune tolerance. Under immune system pressure, Ag loss variants can arise in the targeted tumor cells, leading to relapse. This phenomenon has been observed in T-cell therapies using adoptive transfer of neo-Ag-specific autologous T cells in melanoma patients [48]. Thus, the CPP-linked TAAs cargo has to be as diverse as possible. Tumor cells use various mechanisms to escape immune system pressure; among them is the expression of inhibitory immune checkpoints ligands that negatively regulate T-cell function. Antibodies blocking interaction between these ligands and their receptors, such as anti-CTLA4 (ipilimumab), anti-PD1 (nivolumab, pembrolizumab and pidilizumab) and anti-PD-L1, are currently used clinically with outstanding results on a proportion of patients with aggressive forms of melanoma and several advanced malignancies [81]. Therefore, immune checkpoint inhibition in combination with CPP-based cancer vaccine immunotherapy represents a promising future direction. Finally, careful choice of adjuvant is mandatory to maximize the magnitude of Ag-specific T-cell responses and the resulting anti-tumor effect elicited by CCP-based cancer vaccines.
